# The community voices program to facilitate community–academic researcher partnerships: Stakeholder perspectives on the program’s usefulness

**DOI:** 10.1017/cts.2023.657

**Published:** 2023-10-20

**Authors:** Magaly Ramirez, Jenny Wool, Sonia Bishop, KeliAnne K. Hara-Hubbard, Sou Hyun Jang, Judy Leong, Laurie Hassell, Linda K. Ko

**Affiliations:** 1 Department of Health Systems and Population Health, University of Washington School of Public Health, Seattle, WA, USA; 2 Institute of Translational Health Sciences, Seattle, WA, USA; 3 Department of Sociology, Korea University, Seoul, Korea; 4 VA Puget Sound Healthcare System, Seattle, WA, USA

**Keywords:** Community stakeholders, translational researchers, community-engaged research, qualitative research methods, clinical and translational science award

## Abstract

**Introduction:**

The Institute of Translational Health Sciences (Clinical and Translational Science Awards Program hub) developed a program coined Community Voices to invite communities to submit project ideas and be matched with academic researchers. We describe formative research to understand community and academic researcher perspectives on how the program could facilitate collaborations addressing community priorities.

**Methods:**

We conducted four focus groups with 31 community-based organization (CBO) representatives and 11 semi-structured interviews with academic researchers in the Washington, Wyoming, Alaska, Montana, and Idaho regions. Questions included the appeal of Community Voices to engage community and academic partners, potential program usefulness, and Community Voices’ potential role in building community–academic partnerships. We used an inductive, constant comparison approach to code transcripts and thematic analysis to generate themes.

**Results:**

Most CBO representatives were female (87.1%) and Hispanic/Latino (61.3%). Most academic researchers had a PhD (63.6%) and worked at a university (81.8%). The themes were: (1) community–academic partnerships built on trust will offer mutual benefit, (2) community-initiated project ideas should prioritize community needs, (3) matchmaking will accelerate connections but should not replace time to foster partnership, (4) Community Voices should go beyond matchmaking and provide ongoing support/training, and (5) fostering effective communication is key to partnership success.

**Conclusions:**

Community Voices is a novel, bidirectional community engagement program model that advances current practices of prioritizing researchers’ project ideas. This community-driven program may shift the future direction of community engagement practices where prioritizing community’s ideas becomes the norm of community–academic partnerships in clinical and translational science.

## Introduction

The Clinical and Translational Science Awards (CTSA) Program – established by the National Center for Advancing Translational Sciences (NCATS) at the National Institutes of Health – recognizes the importance of community members as full collaborators in all aspects of clinical and translational research [1,2]. NCATS emphasizes engaging rural, minority, and other underserved populations experiencing a disproportionate burden of conditions. CTSA hubs are expected to develop and implement community engagement strategies based on their unique strengths and the needs of their local environment. Community engagement strategies implemented by CTSA hubs have included providing education and training to prepare researchers and community members to conduct community-engaged research, offering pilot grant funding, and connecting potential partners [3,4]. CTSA hubs have also facilitated community engagement for specific research projects via practice-based research networks (i.e., groups of practicing clinicians and investigators collaborating to answer community-based health care questions and translate research into practice [5]), community advisory boards, consultation services, and by integrating community members in research projects as consultants, staff, and co-investigators [3,6–8]. These CTSA community engagement strategies build capacity among academic researchers and community members and provide resources to cover the costly and time-intensive process of community-engaged research.

Teams conducting clinical and translational research are encouraged to involve community partners in setting the research agenda and planning new projects [9]. In doing so, research is expected to become more focused on health priorities that are important to the community. In practice, however, aligning the health priorities of research institutions and community partners has been identified as a major challenge to community engagement in CTSA hubs [10,11]. Some CTSA hubs have responded to this challenge by working with their community partners to identify areas where community priorities do align with researchers’ interests and expertise, while other CTSA hubs have continued the partnerships despite differing priorities [10]. To achieve NCATS’ goal of accelerating clinical and translational research to address the significant burden of conditions that disproportionately affect rural, minority, and other underserved populations [1], CTSA hubs must develop solutions to address misaligned priorities between research institutions and community partners.

**Figure 1.**
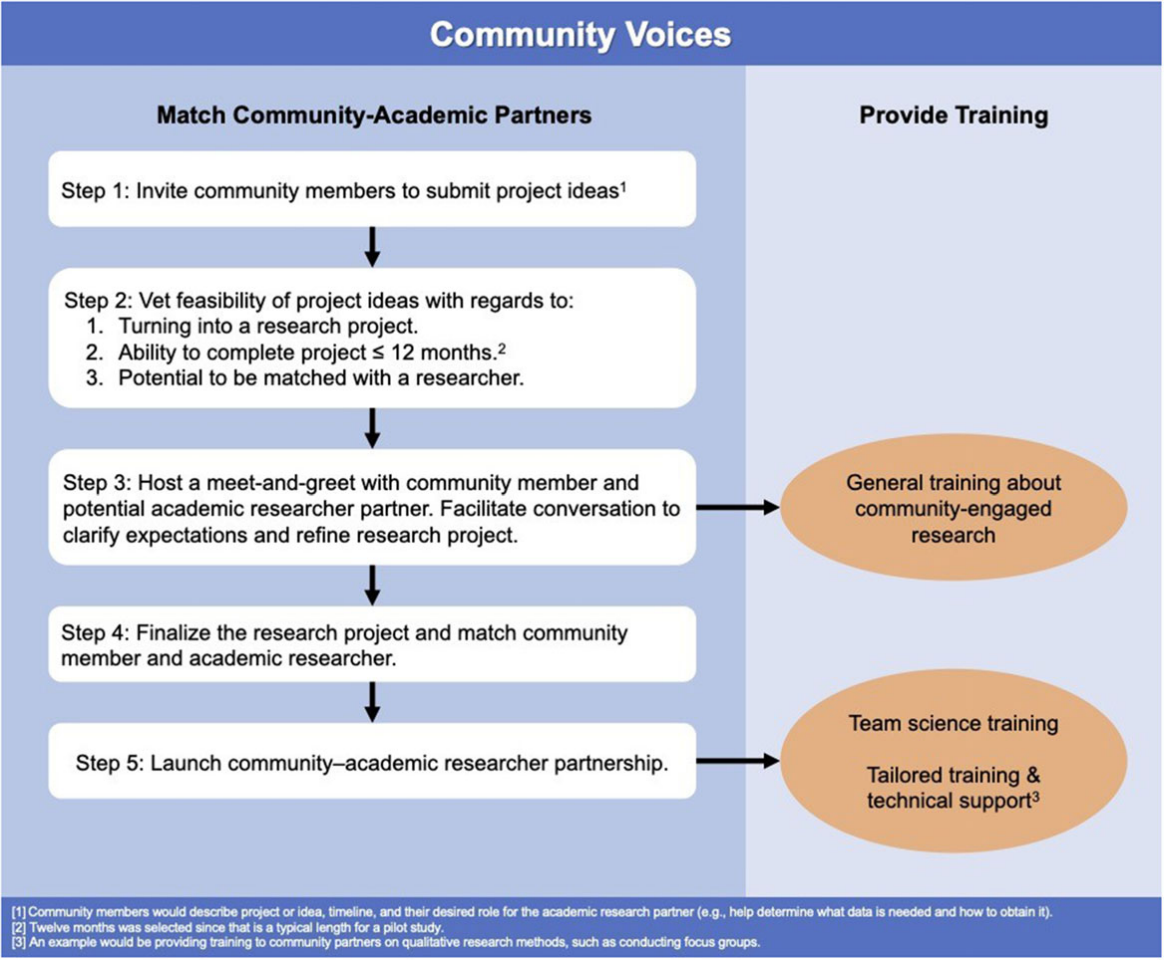
Framework for Community Voices.

The Institute of Translational Health Sciences (ITHS) is a CTSA hub involving the University of Washington, Fred Hutchinson Cancer Center, and Seattle Children’s Hospital. The ITHS Community Engagement core facilitates community–academic researcher partnerships in Washington, Wyoming, Alaska, Montana, and Idaho (WWAMI). In 2017, the Community Engagement core began developing a program coined Community Voices that would invite communities (i.e., community-based organizations, healthcare organizations, and patients) to submit project ideas and be matched with an academic researcher with the same interests to partner on the project. A coordinating center with a director and staff was formed to develop an initial framework that would guide the vision for the Community Voices program. As illustrated in Fig. 1, the vision was for the coordinating center to vet project ideas submitted by community members, match community members with academic researchers, provide training on successful collaboration, and provide ongoing consultation and technical support.

In 2018–2019, while the Community Voices program was still being conceptualized, the coordinating center conducted a qualitative study to elicit feedback from community and academic researchers on the initial framework for Community Voices. This manuscript reports the findings from the qualitative study. The objective is to describe community and academic researcher perspectives on how a program like Community Voices could be useful in facilitating community–academic partnerships to address community priorities. The coordinating center used the qualitative study findings to inform improvements to Community Voices while the program was still under development. In 2020, the program launched when the coordinating center matched the first community and academic research partners.

## Materials and Methods

### Design Overview

We conducted focus groups with representatives from community-based organizations (CBOs) and semi-structured interviews with academic researchers. We recruited representatives from CBOs located in urban and rural counties of Washington state to capture perspectives unique to those geographic areas. We recruited academic researchers residing in the WWAMI region and across academic ranks to capture diverse views based on career experience, geographic diversity, regional research priorities, and resource needs.

### Ethical Approval

The Institutional Review Board at the Fred Hutchinson Cancer Center approved the study. All participants provided verbal informed consent.

### Participant Recruitment

To recruit academic researchers and CBO representatives, a study staff member emailed recipients of an ITHS community–academic partnership award to explain the study’s purpose and invite study participation. ITHS offers these partnership awards to help initiate partnerships between academic and community investigators for new projects [12]. The partnership awards are managed by the ITHS Pilot Awards Program, which is separate from (and established prior to) Community Voices. Partnership award recipients were deemed experienced to understand the purpose of the Community Voices program and provide feedback from both community and academic researchers’ perspectives. We also asked ITHS program leaders to disseminate a recruitment email with information about the study to academic researchers residing in the WWAMI region. If an academic researcher was interested in participating, the study staff member scheduled a phone call to explain the study, answer any questions, and screen for eligibility. An individual was eligible if they held a faculty position at a university in the WWAMI region. Among the 18 academic researchers who were eligible, we randomly selected 12 to participate in an interview (6 junior and 6 senior faculty members). The study staff member obtained verbal informed consent and scheduled an interview. We conducted 11 semi-structured interviews. One faculty member could not be scheduled after multiple attempts.

We also invited CBO representatives that we collaborated with in past studies to participate in the present study. A study staff member emailed CBO representatives to explain the study’s purpose and invite participation in a focus group. The email also asked CBO representatives to share the invitation with other CBO representatives who might be interested in participating. If a CBO representative showed interest, the study staff member scheduled a phone call to explain the study, answer questions, and screen for eligibility. An individual was eligible if they were a chief executive officer, director, or senior manager of a CBO in the WWAMI region. We identified 53 CBO representatives who were interested and eligible to participate. The study staff member obtained verbal informed consent and scheduled participation in a focus group. We conducted four focus groups with 31 CBO representatives. Focus groups had an average of eight participants [range between 4 and 10]. The remaining 22 CBO representatives did not participate: 14 could not be scheduled after multiple attempts and 8 did not show up.

### Setting and Timeline

We began recruiting CBO representatives in October 2019. Between November 2018 and February 2019, we convened two focus groups (with 13 CBO representatives) at Fred Hutchinson Cancer Center in Seattle, Washington (urban focus groups), and two focus groups (with 18 CBO representatives) in a community setting in Toppenish, Washington (rural focus groups). We began recruiting academic researchers in November 2018. Between February and March 2019, we conducted telephone interviews with academic researchers.

### Data Collection

The moderator and interview guides are available in the Supplementary Appendix. The research team, which included a researcher with expertise in community-based participatory research, designed the focus group and interview guides. They also collected input from community partners when designing the guides. The questions were similar to those used to capture formative assessments of a program, including importance, acceptability, and feasibility. Prior to attending a scheduled focus group or interview, we asked study participants to complete a self-administered REDCap survey that asked about their demographic characteristics. The surveys for CBO representatives also included questions about the demographic characteristics of the CBOs’ client population.

During focus groups, the focus group moderator began by asking questions about CBO representatives’ experience with community–academic partnerships. The moderator then described the Community Voices framework and the standard operating procedures and tools that would be available in the program. The moderator asked questions to understand what it would take for CBOs to engage with Community Voices, what would make Community Voices useful, and what role Community Voices could play in helping CBOs build partnerships with academic researchers.

During interviews with academic researchers, the study staff member described the Community Voices framework and the standard operating procedures and tools that would be available in the program. The interview guide covered similar questions asked to CBO representatives, including questions on program engagement, usefulness, and its role in building community–academic partnerships. We asked similar questions to confirm the findings from the focus groups and the semi-structured interviews.

Focus groups lasted 52–72 minutes and interviews lasted 22–45 minutes. We audio-recorded focus groups and interviews. A professional transcription company transcribed the audio recordings verbatim. We provided gift cards to CBO representatives ($35) and academic researchers ($50) who participated.

### Data Analysis

We coded transcripts from focus groups and interviews separately using ATLAS.ti version 7. We used an inductive, constant comparison approach to code the transcripts [13]. We created a set of tentative *a priori* codes based on the interview and focus group guides. The research team followed inductive coding of the transcripts with deductive coding using the *a priori* codes to ensure information from the questions was retained during coding. Using an iterative process, the team met weekly to refine the codebooks by adding, removing, and revising codes to address inter-rater agreements and to compare new and existing data.

We identified themes from the codes by first reviewing the excerpts within each code and identifying tentative themes based on the content [13]. The interrelationship across and within themes was analyzed, resulting in a collection of candidate themes. Next, candidate themes were reviewed by the research team. The themes were refined to ensure excerpts within themes cohered and each final theme was distinct and unique.

## Results

### Characteristics of Study Participants

Table 1 presents the characteristics of CBO representatives who participated in a focus group. Participants represented CBOs located in Western, Central, and Eastern Washington. The CBOs spanned a variety of organization types, including clinics (*n* = 11, 35.5%), social service agencies (*n* = 10, 32.3%), and schools (*n* = 6, 19.4%). Most (*n* = 22, 71.0%) reported that their CBOs had partnered with an academic researcher in the past.

**Table 1.** Characteristics of community-based organization (CBO) representatives

Characteristic of CBO representatives	CBO representatives (*N* = 31)
Age (years), mean (standard deviation)	48.4 (14.2)
Female, *n* (%)	27 (87.1)
Race, *n* (%)	
White	17 (54.8)
Black or African American	5 (16.1)
American Indian or Alaska Native	3 (9.7)
Other	1 (3.2)
Not reported	5 (16.1)
Hispanic/Latino ethnicity, *n* (%)	19 (61.3)
Education^1^ (years), mean (standard deviation)	16.2 (3.2)
Type of health insurance, *n* (%)	
Private	21 (67.7)
Public	8 (25.8)
Other	7 (22.6)
None	1 (3.2)

1 Years of education excludes Kindergarten.

Table 2 presents the characteristics of CBOs’ client population, as reported by CBO representatives who participated in a focus group. All CBOs served populations with income below the 200% federal poverty level; the median percentage of clients in this income group was 85%. Thirty CBOs served Hispanic/Latino populations; the median percentage of Hispanic/Latino clients was 50%. Twenty-four CBOs served populations living in rural regions; the median percentage of clients living in rural regions was 50%.

**Table 2.** Characteristics of the community-based organization (CBO) client population

Demographic group	Number (percentage) of CBOs serving the demographic group	Median percentage of clients in demographic group
Hispanic/Latino	30 (96.8)	50
Non-Hispanic White	29 (93.5)	20
Non-Hispanic Black or African American	25 (80.6)	5
American Indian or Alaska Native	28 (90.3)	3
Asian, Pacific Islander, or Native Hawaiian	24 (77.4)	3
Income below 200% federal poverty level	31 (100)	85
LGBTQ+	21 (67.7)	10
Lives in rural region	24 (77.4)	50

Table 3 presents the characteristics of the academic researchers who participated in an interview. Most had a PhD (*n* = 7, 63.6%) and worked at a university (*n* = 9, 81.8%). The number of researchers was distributed relatively equally across academic ranks. Most researchers were female (*n* = 10, 90.9%) and White (*n* = 9, 81.8%).

**Table 3.** Characteristics of academic researchers

Characteristic	Academic researchers (*N* = 11)
Female, *n* (%)	10 (90.9)
Race	
White, *n* (%)	8 (72.7)
Asian, *n* (%)	1 (9.1)
Two or more races, *n* (%)	2 (18.2)
Hispanic/Latino ethnicity, *n* (%)	0 (0)
Educational attainment	
PhD, *n* (%)	7 (63.6)
MD, *n* (%)	2 (18.1)
Other, *n* (%)	3 (27.2)
Academic title	
Professor, *n* (%)	3 (27.2)
Associate professor, *n* (%)	3 (27.2)
Assistant professor, *n* (%)	3 (27.2)
Other, *n* (%)	2 (18.1)
Organization type	
University, *n* (%)	9 (81.8)
Research institution, *n* (%)	2 (18.1)
Healthcare organization, *n* (%)	2 (18.1)
Other, *n* (%)	1 (9.1)

### Overview of Themes

We identified five themes regarding study participants’ perspectives on the Community Voices program. The themes were: (1) a community–academic partnership built on trust will offer mutual benefit; (2) community-initiated ideas will need to prioritize community needs; (3) matchmaking between CBOs and academic researchers will accelerate connections but should not replace time to foster partnership; (4) Community Voices needs to go beyond matching and provide ongoing support and training; and (5) fostering effective communication will be key to ensuring partnership success. Below, we report the themes and the modifications made to the Community Voices program based on participants’ suggestions.

#### Theme 1: A community–academic partnership built on trust will offer mutual benefit

Participants were generally interested in Community Voices because of the perceived benefits of community–academic partnerships, and they explained how establishing the community’s trust was a necessary step to experiencing these benefits. CBO representatives felt that partnerships with academic researchers helped them achieve their organizational goals. For example, some CBOs expressed desire to apply for grants but lacked the preliminary data required for grant applications and the capacity to undertake a robust data collection project. A CBO representative described needing support in “data collection and analyzing where then I could turn around and possibly have that data for grants. We don’t have a grant department that does that for us” [rural focus group 1, participant 10]. Community Voices was perceived as an opportunity for CBOs to access needed research expertise and build capacity.

Academic researchers shared that community partners provided important perspectives that challenged researchers’ assumptions, made research more applicable to communities, and sparked insights for new research directions. Academic researchers recognized that community partners are needed for all aspects of research and emphasized their value in recruitment and retention of study participants. One academic researcher stated, “I don’t have a hospital attached to my university, so without community partners, I can’t do the kind of research I want to do…you know, patient population” [interview participant 305]. Another academic researcher shared their experience regarding the significance of CBO partnership in gaining broader trust in the community and recruiting study participants:

They really provided community members or potential participants with a sense of trust and that participation would be confidential and that [community members] could really trust [CBOs]. And given that people at these organizations knew potential participants and were connected to the community, they were able to really foster those relationships for me and explain the study to them in ways that really demonstrated that it would be okay to participate and that they didn’t need to worry about all the things that they would’ve had to worry about if I had just approached them without the help of the community. [interview participant 331]

To build a community–academic partnership and experience the benefits, participants acknowledged that establishing the community’s trust is necessary. According to CBO representatives, factors that undermined trust included the underrepresentation of minority researchers, feelings of being over-studied, frequent rotation of researchers, lack of program sustainability, and lack of data sharing. One CBO representative welcomed academic researchers’ interest in seeking community partners to build trust with community members. They said, “Once [academic researchers] get our trust, they got it, you know, with our communities. Once you’ve got it, we can move forward. And then if – then you become part of the community that we can’t wait to see when you come over that hill. I really like that researchers are now seeing that the road comes this way from Seattle just as much as it’s going that way” [rural focus group 1, participant 6]. Participants believed that Community Voices could help foster trust between researchers and communities.

#### Theme 2: Community-initiated ideas will need to prioritize community needs

A feature of Community Voices that resonated with many participants, especially CBO representatives, was that the community would initiate ideas for projects based on their own identified health priorities. According to CBO representatives, academic researchers typically approach them after a grant has already been funded and ask for help recruiting study participants. CBOs further shared that academic researchers traditionally seek minimal community input when determining which health issues to address. CBOs expressed the need to reverse this process. A CBO representative explained the importance of academic researchers involving the community from the start to ensure that the health needs of the community are prioritized:

I think it’s important for community organizations to partner with academic research to…help our community be part of it from the beginning and not just to have the researcher come and decide what are the needs of our community. So, it is important for the researchers to involve our community so that way they address the issues our community are facing but not whatever the researcher wants to address based on their research. [rural focus group 1, participant 5]

Academic researchers stated that differing perspectives on health priorities is a barrier to community–academic partnerships. They acknowledged that academic researchers oftentimes approach the community with a rigid research agenda but instead need to be open to learning what is important from the community’s perspective. One academic researcher shared this sentiment: “I think researchers tend to be so motivated by their specific field of study that if – it’s almost as if you went to the community first and understood what the needs were out there…I think would be a really big step” [interview participant 303].

Academic researchers felt that Community Voices would send the message that community input is valued in determining the focus of research studies. As one academic researcher explained, “What’s attractive about [Community Voices] as you set it up is that you’re actually calling out to those community members and saying, “We want your ideas. You know, your ideas are important,” so I think that’s really a valuable thing” [interview participant 305]. Academic researchers, many of whom had previous experience with community-engaged research, expressed that it was imperative to seek community input from the start. In doing so, they believed community partners would be empowered to help drive the research direction and be encouraged to continue engaging in community–academic partnerships in the future.

Participants perceived that the nontraditional or “backward” way of generating research project ideas in Community Voices would help elevate a community’s health priorities. Both CBO representatives and academic researchers suggested that partnerships formed through Community Voices need to begin with understanding the community’s needs. This step ensures studies are designed and conducted to meet the needs identified by the community.

#### Theme 3: Matchmaking between CBOs and academic researchers will accelerate connections but it should not replace time to foster partnership

Another program feature that resonated with many participants, especially academic researchers, was the matchmaking aspect of Community Voices. When a CBO has an idea for a project, they would contact the coordinating center to be matched with an academic researcher who has shared interests and complementary expertise. Participants shared that this would accelerate the formation of community–academic partnerships. Academic researchers noted the time and energy required to build partnerships with community organizations as a major barrier to conducting community-engaged research. CBO representatives shared the sentiment that building partnerships required a significant amount of time. Community Voices, therefore, was viewed as a potential solution by both CBO representatives and academic researchers. An academic researcher stated:

The reason I like the idea of the Community Voices is…It’s hard. You’ve gotta build a base of trust and respect, mutual respect, and kind of a mutual – not a friendship, but a working relationship, and that takes time. And I think so often, what happens with researchers, especially a junior researcher who is on the tenure timeline, that you can get frustrated and grow impatient. [interview participant 306]

Academic researchers said that participation in Community Voices would streamline the process of connecting with community partners compared to approaching potential community partners on their own. Still, academic researchers recognized that building a genuine partnership takes time and warned of forgoing this fundamental work. One academic researcher explained:

You need to kind of put in that energy and develop those relationships and foster that trust. So, in a sense, [Community Voices] makes it a lot easier and efficient on one end, but it can kinda take away that process where it is this fostering of relationships and demonstrating to the community that you are committed, you’re not just kind of coming in and trying to do this project and trying to find someone to help you reach your own goals. [interview participant 331]

Academic researchers said researchers would benefit from the coordinating center emphasizing the importance of taking the time to develop authentic relationships with community partners. They suggested that during the matching process, the coordinating center should vet academic researchers to ensure their time commitment to build a genuine partnership. Doing so would not only increase the initial project’s likelihood of success but could also result in a long-term, multi-project partnership. One academic researcher, for example, said the ideal outcome of participation in Community Voices would be “having the organization…really excited and happy with the outcomes and wanting to work with us further and wanting to do more with us” [interview participant 321]. They further elaborated that the result would be possible if academic researchers were genuinely interested in the partnership and were willing to invest the time to build it.

#### Theme 4: Community Voices need to go beyond matching and provide ongoing support and training to build capacity and foster partnership success

Many academic researchers suggested that Community Voices play an ongoing role in the community–academic partnership throughout the duration of the project. For example, an academic researcher stated that they would find value in Community Voices if “it was not simply just a matchmaking then leaving. But it was a matchmaking and then some kind of ongoing sort of monitoring engagement, making sure things were going well” [interview participant 315]. During check-ins with community–academic partners, academic researchers suggested that Community Voices act as a mediator to facilitate difficult conversations and conflict resolution.

Study participants suggested that, after a match has been made, Community Voices assist the new partners in creating formal partnership agreements. One of the key elements of the partnership agreement would be how each partner defines success. For CBO representatives, success would mean being treated as an equal partner, participating in educational opportunities, and building organizational capacity. A CBO representative, for example, described that success would be “if the research process has increased the internal capacity of the CBO, so they can do more [research] internally and that they’re left more skilled and more knowledgeable of how those things work, so that when the researcher leaves, it’s not just like, “That was nice, and now we don’t have that,’ but they are building their own capacity” [urban focus group 2, participant 6]. Study participants expressed a desire for Community Voices to play a role in creating a shared definition of success to be included in the partnership agreement, along with the project objectives, timeline, deliverables, data sharing plan, expectations, and partner roles and responsibilities.

Furthermore, CBO representatives and academic researchers perceived that incorporating training opportunities would increase the usefulness of the program. CBO representatives were interested in training related to data collection, data analysis, and grant writing. Academic researchers also suggested numerous training topics: improving communication skills, forming project teams, working in culturally diverse teams, developing budgets, obtaining access to clinical data, developing data sharing agreements, leading effective meetings, and disseminating study findings in the community. In terms of the modality for delivering the training, an academic researcher expressed caution:

I think [the training] would be effective as an actual person rather than as a tool. I think that, you know, just seeing lists of best practices or here’s how to run a meeting or develop an agenda and like, how many of those things can go wrong when implemented poorly. Like, the best ideas can still get implemented in a way that ends up being detrimental rather than helpful to the project. [interview participant 321]

To address potential issues and accommodate different learning styles, academic researchers said Community Voices should consider delivering the training using a combination of modalities. They were interested in self-directed learning (e.g., slideshows, documents) and real-time training sessions led by Community Voices staff.

#### Theme 5: Fostering effective communication will be key to ensuring partnership success

Study participants with previous experience in community-engaged research shared that communication practices influenced partnership success. CBO representatives shared that regularly scheduled meetings with academic researchers helped foster effective communication. They appreciated being up-to-date on all aspects of the project, including research activities, timeline, budget, issues, and next steps. Similarly, CBO representatives believed it would be important for community–academic partners to participate in regular team meetings. One CBO representative suggested frequent meetings for partners to get to know each other, establish project goals, and check in regularly. The CBO representative described a previous partnership with an academic researcher as a successful model of collaboration:

How we worked before which has worked for us is we have a meeting with the researcher, we let them know sometimes even with data we know what the needs are and then collect the data to help steer, then also show direction. So, we do monthly check-ins or weekly until we get to know each other and what we want. That’s very clear at the forefront and then to kind of move towards that…So, I would say that more frequent visits…meet throughout, you know. [rural focus group 1, participant 6]

Furthermore, some CBO representatives expressed discontent with how academic researchers communicate science. If CBO representatives are to partner with academic researchers via Community Voices, there is a desire for academic researchers to become more effective communicators of science (e.g., by minimizing the use of jargon and acronyms). One CBO representative implored academic researchers to use plain language when working in communities that have diverse levels of educational attainment. CBOs further shared that academic researchers’ communication style can be perceived as condescending, diminishing community members’ willingness to contribute their knowledge and expertise. One CBO representative explained their personal experience of communicating with researchers:

I have talked to researchers before and I’m not uneducated, and there were sometimes when I’m just like, I’m not really – can you repeat that and can you please put it in layman’s terms, ‘cause I have no idea. So, when we’re talking about partnering with a community it would be really important for [academic researchers] to remember community as a general rule are not PhDs…otherwise you’re actually going to sit there and I think discourage community members, because if they don’t understand it or they feel like they’re…not being connected to, they’re not gonna want to do it, and that’s a big deal. [urban focus group 1, participant 1]

Some academic researchers also discussed communication challenges with community partners due to differences in education and areas of expertise. They viewed communication as a potential barrier to participation in Community Voices. One academic researcher stated, “I think it would be interesting to see if [Community Voices] works…I don’t know that all community partners necessarily speak the same language. So, literally, academics and research, it’s an entirely different language” [interview participant 309]. The consensus among study participants was that for community–academic partnerships formed through Community Voices to be successful, the partners need to be proactive in addressing communication challenges.

### Modifications Made to the Community Voices Program Based on Participants’ Suggestions

The suggestions from CBO representatives and academic researchers informed four major modifications to Community Voices prior to the launch of the program in 2020. First, we built a robust vetting process for academic researchers using a two-tier mechanism. In the first tier, we assessed interest in being partnered with a community member and experience with community engagement. In the second tier, we assessed commitment to the project and the community. Ultimately, the community members would have the final decision on whether to move forward with the partnership after reviewing the information gathered by the coordinating center. Second, we developed robust training on community-engaged research and processes to foster effective communication for academic researchers. The training on community-engaged research covered what it is, how to address challenges when collaborating with community partners, and how to remain productive while conducting community-engaged research. The training on effective communication included key principles of successful communication among complex teams and co-developing a communication plan. Third, we created a team charter that community–academic research partners could use in their first project meeting to co-design the objectives, shared vision, and implementation plan. Fourth, we provided ongoing assistance to partners, such as providing administrative assistance during meetings and helping navigate conflicts.

## Discussion

We identified five themes in a formative study to understand CBO representative and academic researcher perspectives on how Community Voices could be useful in facilitating community–academic partnerships in the WWAMI region. First, participants described how they would benefit from a community–academic partnership built on trust, including the opportunity to address organizational challenges related to data collection for grant proposals (perceived benefit to CBO representatives) and enhance recruitment and retention of study participants (perceived benefit to academic researchers). Second, participants believed that by enabling community members to initiate project ideas, Community Voices would ensure that community needs were prioritized. Third, participants stated that the process of matching communities and academic researchers would accelerate connections that are typically time-intensive and a barrier to conducting community-engaged research. Fourth, participants suggested that ongoing support from the program’s coordinating center could help with creating partnership agreements, resolving conflict, and training to prepare for the partnership. Finally, participants indicated that the program would be useful if it fostered effective communication practices, which they viewed as essential for successful community–academic partnerships.

Our findings corroborate results from prior studies on the importance of conducting research focused on community-identified health priorities. In particular, the findings align with community engagement literature and discussions highlighting that meaningful community engagement in clinical and translational research involves responding to community priorities [14–22]. For example, to address the misalignment of research priorities between communities and researchers (a major challenge across CTSA hubs [10]), CTSA hubs are encouraged to seek an understanding of community needs and align research priorities accordingly [23]. CTSA hubs have developed strategies to obtain moderate to high levels of community engagement in research by utilizing community advisory boards, expert panels, consultations, or by having community members as staff or co-investigators [3]. Community Voices, however, would be a unique community engagement strategy in that it would create infrastructure for community members to submit project ideas and be matched with academic researchers who share interests and have complementary expertise to move the project forward. In this model, project ideas would be initiated by the community rather than by researchers. Our formative study suggests Community Voices is a promising community engagement strategy to directly address community-identified priorities. Future research will include launching the Community Voices program, providing training to community and academic research partners, and formally evaluating the community–academic research partnerships formed via the program using a mixed methods approach.

Our findings show that CTSA hubs can create a model where project ideas originate from within the community and researchers are recruited to work on projects based on community needs and priorities. Past studies on this type of approach were mainly among researchers conducting CBPR [24–26]. While CBPR has demonstrated meaningful partnerships with communities, it has also been noted as time- and effort-intensive [20,21]. Our study reveals that with a community-centric model, a coordinating center with adequate resources, and robust training, Community Voices can be operationalized into a program that addresses community and researcher needs. Many community engagement approaches by CTSA hubs remain heavily directed by the project needs of academic researchers [3,10]. Our findings show that projects can be made bidirectional by creating a new mechanism for community members to initiate project ideas and a coordinating center to vet project ideas submitted by community members, provide training to matched community–academic partners, and provide ongoing consultation and technical support. Additionally, we believe our formative study seeking early input from potential users of the Community Voices program (namely, CBO representatives and academic researchers) is a promising strategy for improving their acceptance and our feasibility of carrying out the program. While funding for projects was not a salient theme in the qualitative analysis of our formative study, there are ongoing discussions at ITHS about pilot funding to support community–academic partners in carrying out their projects.

Our study has limitations. First, participants did not have direct experience with Community Voices. Perspectives on the program were based on descriptions we provided during focus groups and interviews. Targeted feedback based on direct experience with Community Voices could reveal additional opportunities to increase the program’s usefulness in facilitating community–academic partnerships. Second, several CBO representatives who participated had previously collaborated on research studies and with our research team. This may have influenced how CBO representatives responded to focus group questions – perhaps responses were different and/or more favorable than we would have observed from participants who had no prior research experience or relationship with our research team. Third, academic researchers who participated were likely more interested in community–academic partnerships than those who did not express interest in study participation. Some academic researchers had previous experience with community-engaged research. This limits our ability to understand the perspectives of academic researchers without community engagement experience and whose fields do not traditionally engage communities in research. Future research is needed to understand the perspectives of CBO representatives and academic researchers with no prior experience partnering on research studies.

Community Voices offers an innovative community engagement model to ensure the priorities of community members and academic researchers align and partners are fully prepared for research collaboration. Our formative study found that CBO representatives and academic researchers in the WWAMI region – who represent potential users of Community Voices – were attracted to key elements of the program. Notably, they showed enthusiasm for how Community Voices focused on projects identified by the community, matched community members with an academic researcher to build a partnership that moves projects forward, and provided training and technical support from the coordinating center. This promising model has the potential to address challenges of community engagement in CTSA hubs [4] and thereby advance community engagement practices in clinical and translational research [23,27].

## Supporting information

Ramirez et al. supplementary material Ramirez et al. supplementary material

## Supplementary material

The supplementary material for this article can be found at https://doi.org/10.1017/cts.2023.657.
