# Supplementary material for: The community voices program to facilitate community–academic researcher partnerships: Stakeholder perspectives on the program’s usefulness
Source: J Clin Transl Sci. 2023 Oct 20;7(1):e237. doi: 10.1017/cts.2023.657 (PMC10663767; doi:10.1017/cts.2023.657)
Supplement: Ramirez et al. supplementary material [file S205986612300657Xsup001.docx]

# Overview of the Community Voices Program

The foundation of CV was based on the academic literature on community engagement and community-based participatory research (CBPR), our experience conducting CBPR, our experience working with researchers conducting clinical and translational research, and informal conversation with the community researchers. CV is an innovative platform that will lessen the amount of legwork needed from both researchers and the community by bringing ideas and projects initiated by CBOs to researchers and combining in-person interactions and web-based interfaces.

**At the core of CV is the Coordinating Center which consists of faculty leaders, intake project managers, lead project managers, and other personnel from the CE program.** The faculty leaders are responsible for oversight of CV projects. The intake project managers assume responsibility for incoming projects until a match is successfully made between a CBO and an academic researcher. At that point the lead project managers will take over for the lifespan of those projects.

**To elicit community ideas for projects, the CV Coordinating Center does outreach to CBOs around the WWAMI region that includes community-based practice sites, large healthcare systems, and patients and patient advocacy groups.** The outreach starts with the intake project managers disseminating to CBOs an information sheet that outlines the CV program. CBOs who are interested in strengthening the scientific rigor of their project (e.g., developing a data collection and analysis tool) and implement evidence-based practices to improve community health, are then prompted to click on a URL to a web-based questionnaire that gathers information on the project(s) for which they are looking for an academic researcher. Questions at this level are categorized as Tier 1 which includes a description of the project or idea; project timeline; the type of academic collaboration that they are looking for (e.g., consultation); and current funding, if any (Table 3).

*Supplementary Table*. Community Voices Community Organization Intake Form

| **Name (first, last)**: | **Phone Number**: |
| --- | --- |
| **Organization Name**: | **Email:** |
|  |  |
| **Region:**  *Please indicate where your organization is located or the region that it serves:* | |
|  | |
| **Project Title (if available):** | |
|  | |
| **Please describe your project or idea** | |
|  | |
| **Are there funds to support your request?**  ☐ Yes ☐ No ☐ Unsure | |
|  | |
| **What is the project timeline?**  *For example, grant deadlines, key event dates, projected start date or month. If there is no timeline, please tell us.* | |
|  | |
| **Please describe how your project or idea will benefit your organization and/or community?**  ☐ Leader: researcher is the primary decision maker on the project  ☐ Co-leader: researcher leads as an equal partner with the community leader  ☐ Consultant: researcher helps with a specific part of the study, such as data analysis  ☐ Advisor: researcher provides general input about the project | |
|  | |
| **If you envision another role for your collaborator, or you’re not sure what role he/she will have in your project, please describe what you think the collaborator will do.**  *Examples: -Help to determine what data we need and where we can get it -Provide information about diabetes and why it's so prevalent in our community -Give advice on the grant writing process* | |
| **Publication of research results is important to researchers’ careers. Are you willing to publish the results of your project or ideas in a scientific journal?**  ☐ Yes ☐ No ☐ Unsure | |
|  | |
| **Notes:** | |

Responses received from the questionnaire are crucial as they facilitate the vetting process. Responses are compiled into report form and would be used to (1) identify academic researchers that align with the goals and needs of the CBOs, and (2) determine their interest and availability for a collaboration. Potential academic researchers are identified through the networks of the faculty leaders and are contacted to assess for interest and availability. Academic researchers who are interested will respond to the Coordinating Center and have a chance to ask CBOs tailored and specific questions (i.e., Tier 2 questions). Once an academic researcher seems matchable with a CBO, the information is presented to the CBO. The CBO has an opportunity to ask specific questions about the academic researchers and ultimately make the decision to go forward with the matching process. Then, the Coordinating Center will organize a meeting between CBOs and academic researchers to determine if a partnership between CBOs and academic researchers is feasible. A few of the criteria to determine feasibility by the Coordinating Center include clarity of project objectives, scientific component, scope of work for the academic researcher, and timeframe (approximately 12 months).

After the feasibility meeting, the outcomes could be (1) the partnership is launched (i.e., a successful match), or (2) CBOs are placed into a holding queue and/or (3) academic partners receive general training to enhance their knowledge and skills in community engaged research. After being successfully matched, the CV partnership will receive technical support and tailored team training from the CV program to ensure a strong launch pad for partnership progress is made by teams, and bidirectional communications becomes the norm, until project completion.

# Interview Guide for Academic Researcher Interviews

**PROCESS**

Today we’re going to do two things. First, I will ask you some questions about your experience partnering with a community-based organization or a community member in research. Then, we will show you the community voices program and some materials that will be used for this program. I will then ask for your comments on how to make a useful program for academic researchers and community-based organizations. Any questions?

OK – let’s get started then.

**General questionS**

1. What are your thoughts about partnering with community members to undertake a community health idea?
2. Please tell us about a community-academic project you’ve conducted in the last 5 years. If you’ve worked on more than one project, please choose the project in which you had the most interaction with your community partner.
   1. To what extent were you involved in the community-academic project you described in the previous question? [PROBE: Principal Investigator, Co-investigator, Consultant or subject matter expert (e.g., statistical analysis, trial design, evaluation), Participant recruitment
   2. To what extent was the community partner involved in the project you described in question #2? (select all that apply) [PROBE: Community investigator, community liaison, Advisory board member, Consultant or subject matter expert (e.g., recruitment, retention), Participant recruitment

**ACCEPTABILITY OF THE COMMUNITY VOICES PROGRAM**

**[SHOW AND EXPLAIN THE COMMUNITY VOICES PROGRAM (MATCHING AND TRAINING) USING THE PPT SLIDES AND THE WEB PORTAL]**

1. In general, what are your thoughts about the Community Voices program?
   1. Have you participated in similar programs? If so, what were they?
2. Do you think researchers need a program like this? Why or why not?
3. Do you think community-based organizations need a program like this?
   1. Can you tell me why or why not?
4. Who do you think will most likely use this program? [AR rank and CBOs]
   1. Who would benefit most from the program? The least?
5. Based on what you know about the Community Voices Program, what expectations do you think researchers might have about the program?
   1. Expectations of the community members

**[PROBE: wants to find a COMMUNITY PARTNER immediately, Guaranteed funding, expecting to work with certain COMMUNITY that may not be a good match, etc.]**

- 1. What do you think their response would be if the community voices program wasn’t able to meet their expectations?

1. What do you think researchers who participate in this program would consider a “success”?
   [**PROBE: to connect with a CBO interested in similar topic to undertake a pilot**]
2. What would it take for AR to engage with this program?  **[PROBE: Incentive, FUNDING/stipenD, Specific training]**

**UTILITY OF THE COMMUNITY VOICES PROGRAM**

1. From the perspective of AR, what do you think would make it harder or prevent others from using the Community Voices program?
   **[PROBE: time commitment, staffing requirements, meeting logistics]**

**From the perspective of CBO.**

1. How do you think we can make this program more useful to community-based organizations and AR? What is still missing to make it more useful?
2. How helpful do you think the Community Voices program will be for community-based organizations and AR?

**IMPORTANCE OF THE COMMUNITY VOICES PROGRAM**

1. How important is the Community Voices program to help you build relationships with community members? How important to you the relationships with community members?
2. How important is the Community Voices program to promoting more trust between the community and academic researchers?
3. Do you think the Community Voices program would be helpful for community-based organizations to have greater trust and confidence to participate in research as a research team? How about training topics?

Are you willing to mentor another researcher without experience in community engagement research if he/she is matched to a community partner?

**CLOSING STATEMENT**

That completes the interview. Do you have any other questions, comments, or final thoughts that you would like to share? Thank you again for your participation.

# Moderator Guide for CBO Representative Focus Groups

**PROCESS**

Today we’re going to do two things. First, we will ask you some questions about your organization’s experience partnering with an academic researcher in research. Then, we will show you the community voices program and some materials that will be used for this program. I will then ask for your comments on how to make the program more acceptable and useful to community-based organizations. Any questions?

OK – let’s get started then.

**General questionS**

1. What are your thoughts about partnering with academic researchers to undertake on a community health idea?
2. Has your organization partnered with an academic researcher before on a research project?
   1. Can you tell me what that was like?

**ACCEPTABILITY OF THE COMMUNITY VOICES PROGRAM**

**[SHOW AND EXPLAIN THE COMMUNITY VOICES PROGRAM (MATCHING AND TRAINING) USING THE PPT SLIDES AND THE WEB PORTAL]**

1. In general, what are your thoughts about the Community Voices program?
   1. Has your organization participated in similar programs? If so, what were they?
2. Do you think your organization and other community-based organizations need a program like this?
   1. Can you tell me why or why not?
3. What types of community-based organizations (e.g., social services, community clinics, faith-based organizations) do you think will most likely use this program?
   1. Who would benefit most from the program? The least?
4. Based on what you know about the Community Voices Program, what expectations do you think other community-based organizations might have about the program?

   **[PROBE: wants to find a researcher immediately, Guaranteed funding, expecting to work with certain researcher that may not be a good match, etc.]**
   1. What do you think their response would be if the community voices program wasn’t able to meet their expectations? `
5. What do you think community-based organizations who participate in this program would consider a “success”?
   [**PROBE: to bring their ideas to the researchers, to connect with a researcher interested in similar topic to undertake a pilot**]
6. What would it take for community-based organizations to engage with this program?  **[PROBE: Incentive, FUNDING/stipenD, Specific training]**

**UTILITY OF THE COMMUNITY VOICES PROGRAM**

1. We have talked about a new program today. Would someone summarize your understanding about the community voices program for the group?

**[PLEASE VALIDATE THAT THEY HAVE CAPTURED THESE 4 ESSENTIAL ELEMENTS:**

- **COMMUNITY BRINGS THEIR IDEAS TO THE RESEARCHER VIA A WEB PORTAL.**
- **A CBO OR COMMUNITY MEMBER WILL BE MATCHED WITH AN ACADEMIC RESEARCHER AND OTHERS WILL MOVE TO THE QUEUE UNTIL A MATCH IS FOUND.**
- **TRAINING ON PARTICIPATORY RESEARCH AND TEAM SCIENCE.**
- **THE CE COORDINATING CENTER WILL BE INVOLVED TO PROVIDE TECHNICAL ASSISTANCE TO THE MATCHED TEAMS.]**

**[IF ALL POINTS WERE CAPTURED, THEN MOVE TO THE NEXT QUESTION.**

**[IF ALL POINTS WERE NOT CAPTURED, THEN ASK OTHER FOCUS GROUP MEMBERS TO CHIME IN. THEN SUMMARIZE THE POINTS AND MOVE TO THE NEXT QUESTION.]**

1. Is there anything about the process that is not clear to you? Please describe.
2. Do you understand how to use this program?
3. From the perspective of community-based organizations, what do you think would make it harder or prevent others from using the community voices program?
   **[PROBE: time commitment, staffing requirements, meeting logistics]**
4. How do you think we can make this program more useful to community-based organizations? What is still missing to make it more useful?
5. How helpful do you think the Community Voices program will be for community-based organizations?

**IMPORTANCE OF THE COMMUNITY VOICES PROGRAM**

1. How important is it for your organization, that a community engagement program through the University Washington is interested in collaborating with you on project ideas that are important to your organization?
   **[PROBE: advances our mission, could help with future funding, get more innovative ideas going]**
2. How important is the Community Voices program to help you build relationships with academic researchers? How important to you and your organization are relationships with academic researchers?
3. How important is the Community Voices program to promoting more trust between the community and academic researchers?
4. Do you think the Community Voices program would be helpful for community-based organizations to have greater trust and confidence to participate in research as a research team? How about training topics?

**CLOSING STATEMENT**

That completes the discussion. Does anyone have any other questions, comments, or final thoughts they would like to share? Thank you again for your participation.
